# Erector spinae plane block added to intercostal nerve block versus intercostal nerve block alone for postoperative analgesia in video-assisted thoracoscopic surgery: A randomized controlled trial

**DOI:** 10.1371/journal.pone.0361408

**Published:** 2026-10-27

**Authors:** Hyerim Kim, Dongwook Won, Jee-Eun Chang, Jin-Young Hwang, Jung-Man Lee, Tae Kyong Kim

**Affiliations:** Department of Anesthesiology and Pain Medicine, SMG-SNU Boramae Medical Center, Seoul National University College of Medicine, Seoul, Korea; Faculty of Medicine, New Valley University, EGYPT

## Abstract

**Objective:**

The aim of the study was to investigate whether the addition of an erector spinae plane block (ESPB) to surgical intercostal nerve block (INB) provides additional postoperative analgesic benefits in patients undergoing video-assisted thoracoscopic surgery (VATS).

**Methods:**

This prospective, randomized controlled trial included 60 adult patients undergoing elective three-port VATS. Patients were randomly assigned to receive either combined ESPB plus INB (EI) or INB alone. Patients, surgeons, ward nurses, postoperative outcome assessors, and data analysts were blinded to group assignment, whereas the anesthesiologists performing the intervention were unblinded. The primary outcome was pain intensity at 1 hour postoperatively, assessed using the Numerical Rating Scale (NRS). Prespecified secondary outcomes included repeated postoperative NRS pain assessments through 48 hours and cumulative opioid consumption at 24 hours.

**Results:**

The NRS pain score at 1 hour postoperatively was 5.83 ± 2.86 in the EI group and 5.52 ± 3.10 in the INB group (mean difference 0.31, 95% confidence interval [CI], −1.25 to 1.87, p = 0.691). In a post hoc exploratory longitudinal analysis, a linear mixed-effects model demonstrated no significant group-by-time interaction in postoperative NRS pain scores (F[7, 401.2] = 0.81, p = 0.581). Cumulative opioid consumption at 24 hours, expressed as intravenous morphine equivalents, was 41.0 mg (interquartile range [IQR], 31.0–58.5) in the EI group and 40.0 mg (IQR, 29.1–48.3) in the INB group (between-group difference, 3.0 mg; 95% CI, −6.2 to 12.0; p = 0.501).

**Conclusions:**

Adding single-injection ESPB to surgical INB did not significantly reduce 1-hour postoperative pain scores or 24-hour opioid consumption following VATS. Larger adequately powered studies are required to determine whether smaller clinically relevant incremental benefits exist.

**Trial registration:**

ClinicalTrials.gov NCT03949790

## Introduction

Postoperative pain after thoracic surgery can lead to pulmonary complications, including increased oxygen consumption, atelectasis, and pneumonia [1,2]. Although video-assisted thoracic surgery (VATS) has demonstrated reduced postoperative pain and accelerated recovery compared to thoracotomy, patients often experience moderate pain, particularly in the immediate postoperative period [3–5]. Opioids, while commonly used for pain management in VATS [3], have dosage limitations due to adverse effects such as nausea, vomiting, respiratory depression, and potential for addiction [3,6]. Consequently, additional regional analgesic techniques are needed for VATS patients.

While epidural blocks have traditionally been employed for pain management following thoracic surgery, their associated risks and technical challenges have led to increased interest in the erector spinae plane block (ESPB) [7]. Since its introduction in 2016, ESPB has been used across various surgical disciplines due to its simplicity, safety, and adaptability [8,9]. However, inconsistent findings and potential publication bias necessitate further studies to establish its efficacy in thoracic surgery [10–12].

Intercostal nerve block (INB) is a standard analgesic technique in thoracic surgery that provides localized pain relief [13]; however, its effectiveness is limited to a focally targeted area because it primarily blocks the ventral rami [13,14]. Considering the potential for broader analgesic coverage with ESPB, which may reach dorsal rami and the paravertebral space to provide posterior and multi-dermatomal analgesia, the addition of ESPB to standard surgical INB may further improve postoperative pain control. However, whether ESPB provides incremental analgesic benefits when surgical INB is already employed remains unclear, and few studies have specifically addressed this question. Thus, we assessed the incremental benefit of adding ESPB to INB in VATS patients by comparing postoperative pain scores and opioid consumption between the two groups.

## Methods

### Patient selection

The Institutional Review Board of Boramae Medical Center (approval no. 30–2019–29) approved the study protocol, and it was registered at ClinicalTrials.gov (NCT03949790) on May 14, 2019. This prospective randomized trial was conducted from June 18, 2019 to June 3, 2020. The study adhered to the guidelines of Good Clinical Practice, and written informed consent was obtained from 60 adult patients who underwent elective VATS with three ports. Patients with severe renal impairment, history of coronary artery disease, cerebral artery stenosis, coagulopathy, thoracic spine abnormalities, skin infection at the procedure site, or prior thoracic surgery were excluded. The first four of these clinical exclusion criteria were specified in the IRB-approved protocol before recruitment but were inadvertently omitted from the ClinicalTrials.gov registry entry.

### Randomization

Patients were randomly allocated to the combined ESPB and INB (EI) group or the INB group using a computer-generated block randomization sequence. To reduce predictability and enhance allocation concealment, block randomization with randomly varying block sizes (2 or 4) was employed, each ensuring a 1:1 allocation ratio. The randomization sequence was computer-generated prior to enrollment using R software by an independent researcher who was not involved in patient recruitment, clinical care, or outcome assessment. Group assignments were concealed in sequentially numbered, opaque, sealed envelopes. These envelopes were opened immediately prior to the anesthesia induction by a nurse who was not involved in subsequent study procedures, postoperative care, or outcome assessment. To maintain blinding to treatment allocation, the surgeon and the nurse responsible for postoperative pain assessment were not present in the operating room while the anesthesiologists performed the study intervention in both groups. Patients, surgeons, ward nurses, postoperative outcome assessors, and data analysts were blinded to group assignment throughout the study. Patients were unaware of whether they received an ESPB in addition to INB. Due to the nature of the nerve block procedure, the attending anesthesiologists performing the intervention were unblinded; however, they were completely excluded from patient postoperative care, outcome evaluation, and data analysis.

### Study protocol

Upon entry into the operating room, patients underwent standard monitoring via three-lead electrocardiogram, pulse oximetry, and blood pressure monitoring. General anesthesia was induced with propofol (1–2 mg/kg) and a target-controlled infusion of remifentanil at an effect-site concentration of 3 ng/mL. Rocuronium (0.6–0.8 mg/kg) was administered to facilitate tracheal intubation. A radial artery catheter was placed for arterial pressure monitoring and blood gas analysis. Endotracheal intubation was performed with a left double-lumen tube. Subsequently, patients were positioned in the lateral decubitus with elevation of the surgical field.

A single-injection ESPB was performed at the T7 vertebral level in the EI group [15]. Ultrasound guidance was employed using a linear L12–3 probe on a CX50 ultrasound system (Philips Healthcare, Andover, MA, USA) positioned approximately 3 cm lateral to the midline. A paramedian sagittal scan identified the erector spinae muscle and the T7 transverse process [15]. Under real-time ultrasound visualization, an 18-gauge, 8 cm Tuohy needle (Perifix, B. Braun Melsungen AG, Melsungen, Germany) was advanced until contacting the T7 transverse process. Correct needle placement was confirmed by injecting a 2–3 mL saline test dose. Subsequently, 30 mL 0.375% ropivacaine was injected into the interfascial plane between the erector spinae muscle and the T7 transverse process. Successful technical placement of ESPB was defined by linear spread of local anesthetic beneath the erector spinae muscle on ultrasound imaging.

Thoracoscopic surgery was performed using a three-port technique. A 3–5 cm utility incision was made in the fifth intercostal space at the anterior axillary line, and two additional 10-mm thoracoscopic ports were placed at two of the sixth, seventh, and eighth intercostal spaces at the posterior axillary line, depending on the surgical approach. At the end of the surgical procedure, surgeons blinded to group allocation performed INBs under direct thoracoscopic visualization in both groups. A 23-gauge needle was advanced subpleurally into the space between the innermost intercostal muscle and the parietal pleura at the targeted intercostal levels. Ropivacaine 0.375% was injected at three intercostal levels corresponding to the port locations: 4 mL at the fifth intercostal space and 3 mL at each of the two additional port levels selected from the sixth, seventh, and eighth intercostal spaces according to the port locations. Technical performance of the blocks was confirmed by observing pleural displacement in real time during thoracoscopy.

A chest tube was inserted through the largest incision for postoperative air drainage. Patients were extubated and transferred to the surgical intensive care or post-anesthesia care unit. A patient-controlled analgesia (PCA) pump (AutoFuser pump, ACE Medical Co., Ltd., Seoul, Korea) was connected intravenously and initiated at the end of the surgery. The pump, with a total volume of 100 mL, contained fentanyl (800 μg for patients ≤ 70 years, 500 μg for patients >70 years), morphine 20 mg, ketorolac 150 mg, and ramosetron 0.3 mg. PCA settings included a basal infusion of 1.0 mL/h, a bolus dose of 1 mL, and a lockout interval of 15 min. Total opioid consumption was calculated from the amounts of fentanyl and morphine delivered via PCA and expressed as intravenous morphine equivalents (mg), using the conversion ratio of 100 μg intravenous fentanyl to 10 mg intravenous morphine [16]. Ketorolac suppositories 15 mg were administered as rescue analgesia for pain unrelieved by PCA.

Independent nurses blinded to the study assessed postoperative pain intensity using a Numerical Rating Scale (NRS) at 1, 2, 3, 4, 5, 6, 24, and 48 hours postoperatively (0 = no pain, 10 = extreme pain). Postoperative sensory testing was not performed to confirm the clinical extent of sensory blockade. Patients were instructed to use the patient-controlled bolus for resting NRS scores >3 or as needed. The PCA pump was primarily patient-controlled; however, ward nurses supervised its administration. Patients were instructed to perform deep breathing exercises using a three-ball incentive spirometer, with volumes recorded. The CONSORT checklist and the study protocol are provided as S1 Checklist and S2 Protocol, respectively.

### Statistical analysis

The primary outcome was the NRS pain score at 1 hour postoperatively. Although the trial registry entry labeled the pain metric as Visual Analogue Scale (VAS), pain scores were collected using the 11-point Numerical Rating Scale (NRS, 0–10). Prespecified secondary outcomes included NRS pain scores at 6, 12, 24 and 48 hours postoperatively, and cumulative opioid consumption at 24 hours. Non-prespecified outcomes were analyzed as post hoc exploratory outcomes and included additional NRS assessments at 2, 3, 4, and 5 hours, the maximum NRS score within the first 24 hours, cumulative opioid consumption at 48 hours, incentive spirometry volumes at 6, 24, and 48 hours, and rescue analgesic use within 48 hours. Although the 12-hour NRS assessment was listed as a prespecified secondary outcome in the trial registry, it was not collected and was therefore unavailable for analysis. Procedure-related adverse events, including pneumothorax, local anesthetic toxicity, respiratory depression, patient reintubation, or cardiac arrest, were also recorded. The prospective sample-size calculation was based on a previous study reporting a mean maximum NRS pain score of 6.16 ± 1.95 within the first 24 postoperative hours in patients undergoing VATS with intercostal nerve block [14]. A sample size of 27 patients per group was calculated to provide 80% power to detect a 25% reduction in this outcome at a two-sided significance level of 0.05. To account for anticipated dropouts, 30 patients were enrolled in each group.

Participants were analyzed according to their randomized treatment assignment. Missing outcome measurements were not imputed. The primary outcome analysis used available-case data from the 1-hour NRS assessment, whereas the longitudinal mixed-effects model included all available repeated NRS measurements. Continuous variables are presented as mean ± standard deviation or median (interquartile range [IQR]), and categorical variables as numbers (percentages). Statistical hypothesis testing of baseline characteristics was intentionally omitted, consistent with recommendations against such testing in randomized trials [17]. Data normality was assessed using the Shapiro-Wilk test and visual inspection of Q-Q plots. Normally distributed continuous variables were compared using Student’s t-test, whereas non-normally distributed continuous variables were compared using the Mann-Whitney U test. For outcomes analyzed using the Mann–Whitney U test, between-group effects were summarized using Hodges–Lehmann location-shift estimates with 95% confidence intervals. Categorical variables were compared using the chi-square test or Fisher’s exact test, as appropriate. Unless otherwise specified, all between-group effect estimates are reported as EI minus INB. Longitudinal NRS pain scores were analyzed using a random-intercept linear mixed-effects model (LMM) fitted by restricted maximum likelihood with Kenward-Roger degrees-of-freedom adjustment. Fixed effects included treatment group, time as a categorical factor, and the group-by-time interaction. Within-participant correlation among repeated measurements was modeled through the participant-specific random intercept. The primary 1-hour between-group comparison was analyzed separately using Student’s t-test and was not adjusted for multiplicity. Post hoc between-group contrasts at individual time points derived from the LMM were adjusted for multiple comparisons using the Holm method. Treatment effects are reported as point estimates with 95% confidence intervals. Model assumptions were assessed by visual inspection of Q-Q plots and residual-versus-fitted plots. Because NRS scores are bounded discrete outcomes, a sensitivity analysis was performed using a generalized estimating equation with an exchangeable working correlation structure and robust sandwich standard errors, including the same fixed effects as the LMM. A two-tailed *p*-value < 0.05 was considered statistically significant. Statistical analyses were performed using R (version 3.5.1; R Foundation for Statistical Computing, Vienna, Austria) and Python (version 3.7; Python Software Foundation, Wilmington, DE, USA).

## Results

### Patients

A total of 60 patients were randomly assigned to the EI group or the INB group. No participants withdrew or were lost to follow-up, and no technical failures of ESPB placement were observed in the EI group (Fig 1). Baseline demographic and perioperative characteristics, including surgical type, duration of surgery, and intraoperative remifentanil use, are summarized in Table 1.

**Table 1:** Patient demographic data.

	EI (n = 30)	INB (n = 30)
Sex (male/female)	19 (63.3%)/ 11 (36.7%)	19 (63.3%)/ 11 (36.7%)
Age	66.2 ± 9.6	68.8 ± 10.6
Height (cm)	161.8 ± 8.7	161.8 ± 8.0
Weight (kg)	62.5 ± 9.2	63.3 ± 8.8
ASA classification (1/2/3)	3 (10.0%)/ 16 (53.3%)/ 11 (36.7%)	2 (6.7%)/ 15 (50.0%)/ 13 (43.3%)
Duration of surgery (minute)	146.7 ± 64.1	145.0 ± 67.5
Intraoperative remifentanil usage (μg)	1170 ± 720	930 ± 410
Type of surgery (lobectomy/ wedge resection/ segmentectomy/ bilobectomy)	20 (66.7%)/ 9 (30.0%)/ 1 (3.3%)/ 0 (0%)	16 (53.3%)/ 9 (30.0%)/ 4 (13.3%)/ 1 (3.3%)

Data are presented as mean ± standard deviation and number (proportion). EI, combined erector spinae plane and intercostal nerve block; INB, intercostal nerve block; ASA PS, American Society of Anesthesiologists physical status

**Fig 1:**
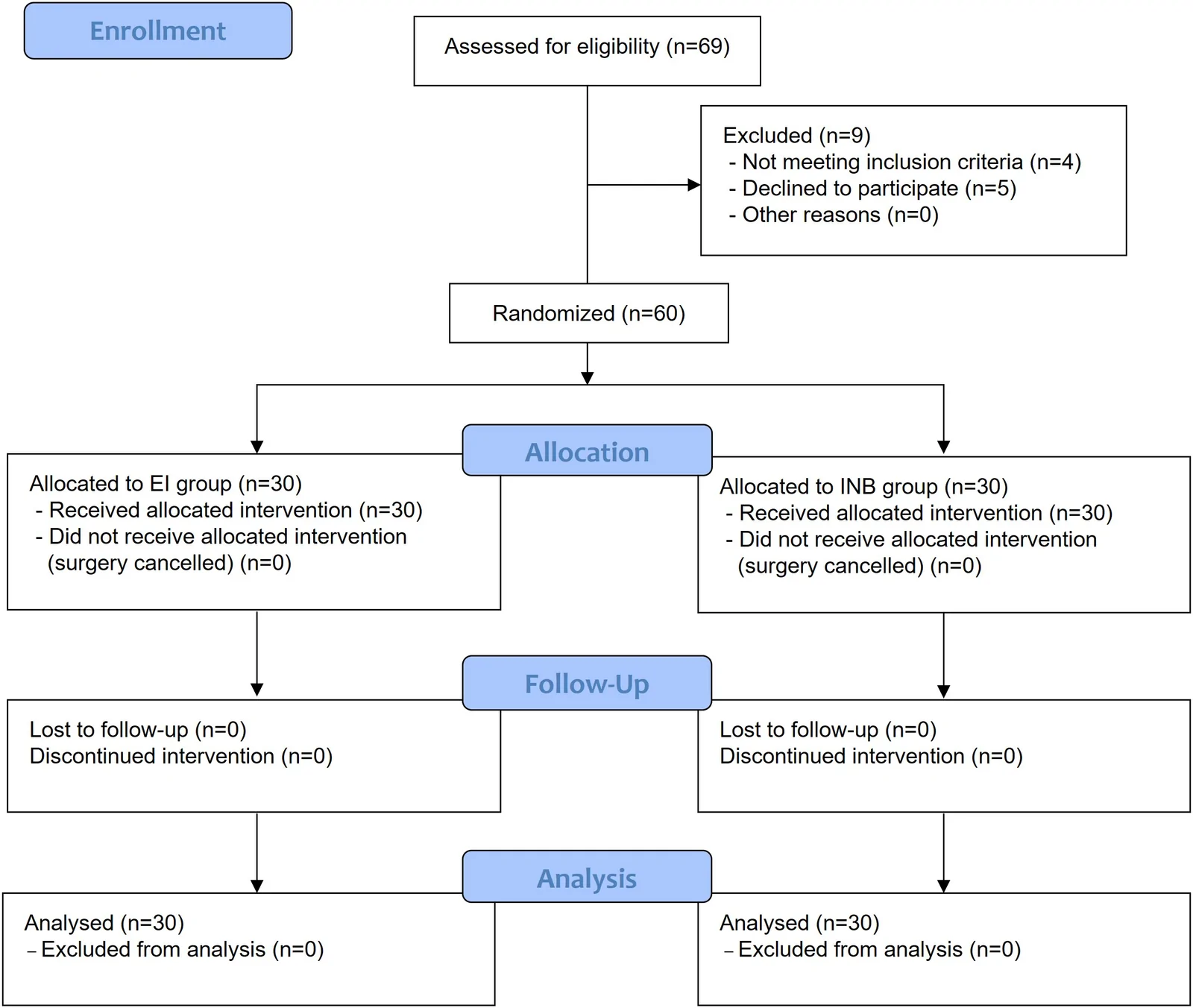
CONSORT diagram. EI: combined erector spinae plane and intercostal nerve block; INB: intercostal nerve block.

### Postoperative pain score

The NRS pain score at 1 hour postoperatively was 5.83 ± 2.86 in the EI group and 5.52 ± 3.10 in the INB group, showing no statistically significant difference (mean difference: 0.31, 95% CI: −1.25 to 1.87, p = 0.691) (Fig 2). One participant in the EI group had a missing 1-hour NRS measurement. Mean postoperative NRS pain scores were highest at 1 hour and gradually subsided over the subsequent 48 hours in both groups. In a post hoc exploratory analysis, the maximum NRS score within the first 24 hours was 6.13 ± 2.67 in the EI group and 6.90 ± 2.43 in the INB group (mean difference: −0.77, 95% CI: −2.08 to 0.55, p = 0.249). In a post hoc exploratory longitudinal analysis using all available repeated NRS measurements, the linear mixed-effects model demonstrated no significant group-by-time interaction in postoperative NRS pain scores (F[7, 401.2] = 0.81, p = 0.581), providing no statistical evidence that the temporal pattern of postoperative pain differed between the groups. Time-specific between-group contrasts derived from the LMM are presented in S3 Table. A sensitivity analysis using robust GEE yielded a similar result (Wald χ² [7] = 7.36, p = 0.392).

**Fig 2:**
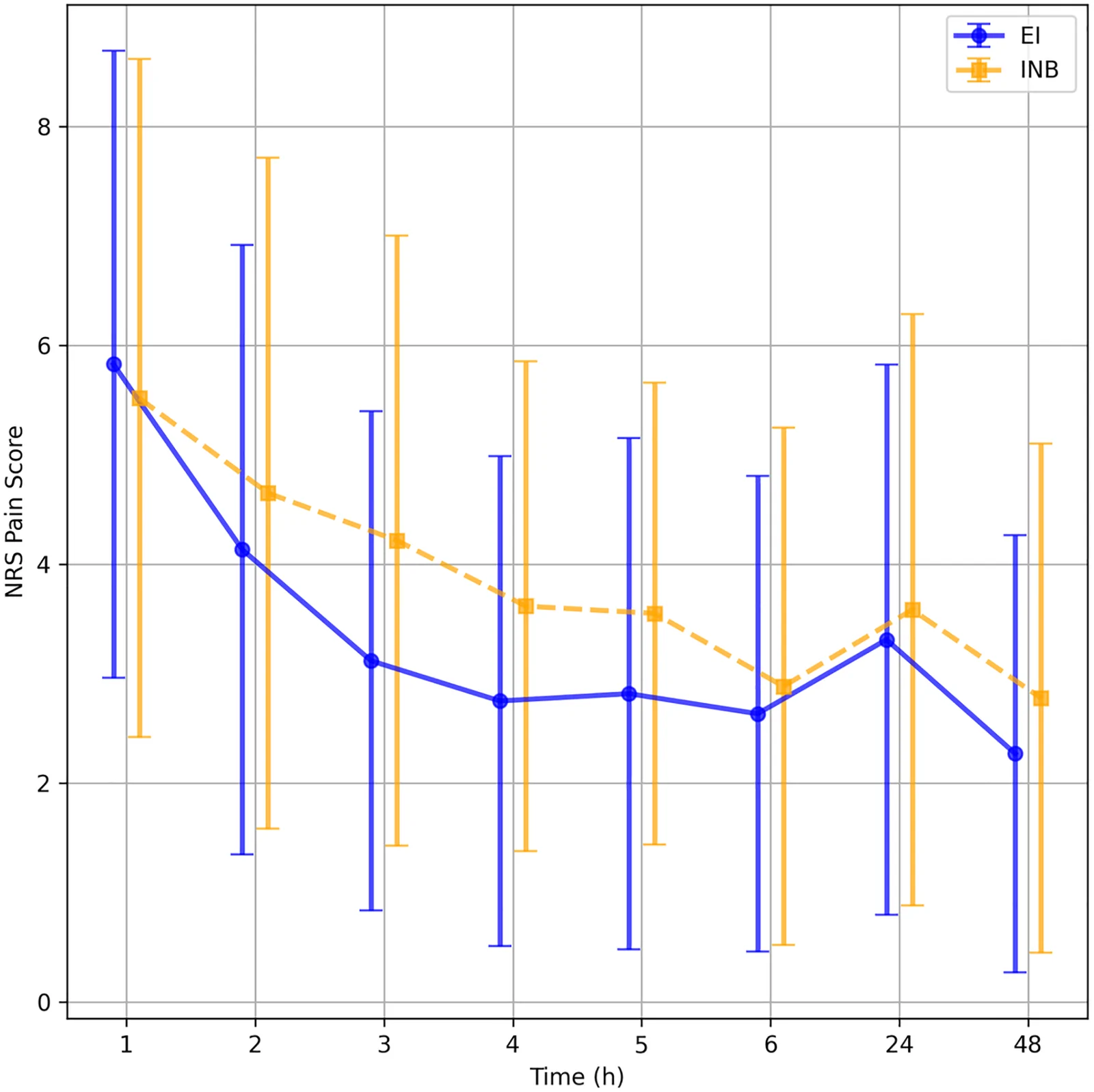
Pain score after surgery. NRS: Numerical Rating Scale; EI: combined erector spinae plane and intercostal nerve block; INB: intercostal nerve block.

### Postoperative opioid consumption

Cumulative postoperative opioid consumption at 24 hours, expressed as intravenous morphine equivalents, did not differ significantly between the groups (between-group difference: 3.0 mg, 95% CI: −6.2 to 12.0, p = 0.501). In a post hoc exploratory analysis, cumulative opioid consumption at 48 hours also did not differ significantly between groups (between-group difference: 0.0 mg, 95% CI: −10.0 to 11.6, p = 0.976) (Table 2). Rescue analgesia within 48 hours, also analyzed as a post hoc exploratory outcome, was required by 20% of patients in the EI group and 40% in the INB group (risk difference: −20.0 percentage points, 95% CI: −40.6 to 3.2, p = 0.091).

**Table 2:** Cumulative postoperative opioid consumption via patient-controlled analgesia.

	EI	INB	Effect estimate (95% CI)	P-value
0-24 h, intravenous morphine equivalents (mg)	41.0 (31.0–58.5)	40.0 (29.1–48.3)	3.0 (−6.2 to 12.0)	0.501
0-48 h, intravenous morphine equivalents (mg)	70.0 (61.0–80.0)	70.0 (64.7–80.0)	0.0 (−10.0 to 11.6)	0.976
Use of rescue analgesics within 48 hours (count)	6 (20%)	12 (40%)	−20.0 percentage points (−40.6 to 3.2)	0.091

Data are presented as median (IQR) and number (proportion). Effect estimates for opioid consumption are Hodges–Lehmann location-shift estimates, whereas the effect estimate for rescue analgesic use is the absolute risk difference, expressed in percentage points. EI, combined erector spinae plane and intercostal nerve block; INB, intercostal nerve block; IQR, interquartile range

### Incentive spirometry

In exploratory analyses, incentive spirometry volumes increased progressively over time at 6, 24, and 48 hours postoperatively in both groups. However, no significant between-group differences were observed at any time point (Table 3).

**Table 3:** Incentive spirometry volume.

	EI	INB	Mean Difference (95% CI)	P-value
Postoperative 6 hours (mL)	773.9 ± 408.0	683.7 ± 580.2	90.2 (−290.8 to 471.1)	0.632
Postoperative 24 hours (mL)	939.1 ± 530.0	896.9 ± 438.5	42.2 (−236.2 to 320.6)	0.762
Postoperative 48 hours (mL)	1067.1 ± 494.3	1052.1 ± 549.6	15.1 (−301.1 to 331.2)	0.924

Data are presented as mean ± standard deviation. EI, combined erector spinae plane and intercostal nerve block; INB, intercostal nerve block.

### Procedure-related complications

No prespecified procedure-related adverse events, including local anesthetic systemic toxicity, pneumothorax, respiratory depression, reintubation, or cardiac arrest, were observed in either group. No patients required conversion to open thoracotomy or early reoperation within the first 48 hours postoperatively.

## Discussion

In this randomized controlled trial, adding ESPB to surgical INB did not significantly reduce postoperative pain scores or opioid consumption following VATS. Specifically, the NRS pain score at 1 hour postoperatively did not differ significantly between the groups. The confidence interval around the primary treatment effect reflected substantial statistical imprecision; therefore, the nonsignificant result should be interpreted as inconclusive rather than as evidence of equivalence. In the longitudinal analysis, there was no statistically significant group-by-time interaction in postoperative NRS scores over 48 hours. Overall, the study did not provide statistically significant evidence of an incremental analgesic effect of single-injection ESPB when surgical INB was already performed.

Previous studies have reported inconsistent findings on the efficacy of ESPB in thoracic surgery, with some demonstrating significant analgesic benefits [18–20]. Several studies have investigated the potential of ESPB as a replacement for other regional nerve blocks, such as paravertebral block, serratus anterior plane block, or intercostal nerve block, for postoperative pain management in thoracic surgery patients [21–24]. A meta-analysis by Koo et al. [10] suggested that ESPB might be a valuable option for managing postoperative pain following thoracic surgery. Unlike other thoracic regional blocks, ESPB may spread across multiple intercostal levels and potentially involve the dorsal and ventral rami of spinal nerves and sympathetic fibers, although the extent and pattern of spread are variable [25]. This suggests a potential synergistic effect when combined with other nerve blocks. However, few studies have investigated the additive analgesic effects of ESPB when surgical INB was already performed. Therefore, our study addresses an important clinical question.

The longitudinal analysis demonstrated no significant group-by-time interaction, providing no statistical evidence that the temporal pattern of postoperative pain differed between groups. Similarly, the maximum NRS score within 24 hours did not differ significantly between groups; however, as a post hoc exploratory outcome reflecting only a single worst pain measurement, it may have been disproportionately influenced by the early postoperative pain peak and may not fully characterize postoperative analgesia over time. The lack of a significant difference in postoperative pain scores between the two groups may be explained by overlapping sensory coverage provided by the two block techniques. A properly performed surgical INB may provide extensive coverage of the anterior and lateral chest wall, leaving little room for additional analgesic benefit from ESPB. The lack of a significant difference may also be attributable to the variable efficacy of ESPB [26,27]. Nielsen et al. [26] demonstrated variable spread of methylene blue during ESPB, indicating inconsistent block effectiveness among patients. These findings suggest that the analgesic effects of ESPB may vary considerably among patients, which may partly account for the absence of a consistent incremental benefit in this study.

In an exploratory analysis, rescue analgesic use was numerically lower in the EI group than in the INB group (20% vs. 40%, p = 0.091); however, this difference did not reach statistical significance and should be interpreted cautiously as a hypothesis-generating finding. Additionally, more patients in the EI group reported complete pain relief (NRS 0) at 48 hours postoperatively than in the INB group (6 vs. 3 patients). Given the exploratory nature of these observations and the small number of events, they should not be interpreted as evidence that ESPB provides analgesia beyond the expected duration of INB [28].

Our study had several limitations. First, the single-center design and relatively small sample size may have limited the generalizability and the statistical precision of the effect estimates. In particular, the study was not powered to detect small incremental treatment effects or uncommon procedure-related adverse events. Furthermore, the study was not designed to assess non-inferiority between the two techniques. Larger multicenter studies are needed to determine whether smaller clinically relevant incremental benefits of adding ESPB to standard surgical INB exist. Second, the reliance on the NRS pain score as the primary outcome measure may have limited the assessment of pain; alternative pain assessment tools, such as detailed pain questionnaires or objective assessments of pain sensitivity, could provide a more comprehensive understanding of pain outcomes [29]. Furthermore, validated quality of recovery scales, such as the QoR-15 questionnaire, were not implemented. The absence of these objective recovery metrics limits the evaluation of the broader clinical impact of the combined analgesic approach. Third, the investigation of single-injection ESPB may not fully capture the potential analgesic benefits of continuous ESPB, which could offer prolonged pain relief [30]. In addition, variation in ESPB-related factors, including drug selection, injection volume, and technique, could have influenced the study outcomes [31,32]. Fourth, although PCA pumps were universally administered for ethical reasons, the specific drug regimen and settings may have confounded the study outcomes. The omission of long-acting non-opioid adjuvants may have partly explained the inadequate early postoperative analgesia and the high NRS scores observed at 1 hour in both groups, and incorporating these agents warrants further investigation to optimize early postoperative pain control. Fifth, postoperative sensory dermatome mapping was not performed; therefore, although technical block performance was assessed by visualization of injectate spread, clinical extent of sensory block could not be confirmed. Sixth, some exclusion criteria specified in the IRB-approved protocol were not included in the public trial registry. This discrepancy should be considered when interpreting the study population and external validity. Seventh, although the registered primary endpoint was the 1-hour postoperative pain score, the prospective sample-size calculation was based on the maximum NRS score within the first 24 postoperative hours. Thus, the prospective power calculation was not specifically based on the registered primary endpoint. In addition, the prespecified 12-hour NRS assessment was not collected, representing a deviation from the registered outcome-assessment schedule. This time point frequently fell around midnight or in the early hours after midnight, making standardized assessment difficult without disrupting patients’ sleep. Consequently, the prespecified 12-hour comparison could not be performed.

## Conclusions

In this randomized trial, adding single-injection ESPB to surgical INB did not significantly reduce 1-hour postoperative pain scores or 24-hour opioid consumption following VATS. Larger adequately powered studies are required to determine whether smaller clinically relevant incremental benefits exist.

## Supporting information

S1 Checklist CONSORT checklist. (DOCX)

S2 Protocol Study protocol approved by the Institutional Review Board. (PDF)

S3 Table Time-specific between-group contrasts in NRS pain scores from the linear mixed-effects model. (DOCX)

S4 Dataset The de-identified individual participant-level dataset. (XLSX)
