# Supplementary material for: Erector spinae plane block added to intercostal nerve block versus intercostal nerve block alone for postoperative analgesia in video-assisted thoracoscopic surgery: A randomized controlled trial
Source: PLoS One. 2026 Oct 27;21(10):e0361408. doi: 10.1371/journal.pone.0361408 (PMC13712513; doi:10.1371/journal.pone.0361408)
Supplement: Supplementary file 1 [file pone.0361408.s002.pdf]

Trial Protocol

Erector spinae plane and intercostal nerve block versus intercostal nerve block alone for postoperative analgesia in video-assisted thoracoscopic surgery: A randomized controlled trial

Clinicaltrials.gov/study/NCT03949790

Study Title

Erector Spinae Plane Block Combined With Intercostal Nerve Block in Video-Assisted Thoracoscopic Surgery: A Randomized Study

Study Design

This study will be a prospective, single-center, randomized controlled trial conducted at Seoul Metropolitan Government-Seoul National University Boramae Medical Center. The study will adhere to the principles of Good Clinical Practice, and written informed consent will be obtained from all participants before enrollment.

Study Population

Adult patients undergoing elective three-port video-assisted thoracoscopic surgery (VATS) will be screened for eligibility.

Inclusion Criteria

- Age ≥20 years.
- Elective VATS

Exclusion Criteria

- Severe renal impairment.
- History of coronary artery disease.
- History of cerebral artery stenosis.
- Coagulopathy.
- Thoracic spine abnormalities.

- Skin infection at the planned procedure site.
- Previous thoracic surgery on the affected side.
- Failure to perform surgical intercostal nerve block.

Randomization and Blinding

Patients will be randomly assigned in a 1:1 ratio to either the combined erector spinae plane block plus intercostal nerve block group (EI group) or the intercostal nerve block alone group (INB group).

Randomization will be performed using a computer-generated block randomization sequence with randomly varying block sizes of 2 or 4. The sequence will be generated by an independent researcher who will not be involved in patient recruitment, clinical care, or outcome assessment. Allocation concealment will be achieved using sequentially numbered, opaque, sealed envelopes.

Patients, surgeons, and investigators responsible for postoperative assessments will remain blinded to group allocation. Because of the nature of the intervention, anesthesiologists performing the blocks will be aware of group allocation but will not participate in postoperative follow-up or outcome assessment.

Perioperative Management

Standard monitoring will include electrocardiography, pulse oximetry, and blood pressure monitoring. General anesthesia will be induced with propofol (1–2 mg/kg), remifentanil target-controlled infusion at an effect-site concentration of 3 ng/mL, and rocuronium (0.6–0.8 mg/kg). A radial arterial catheter will be inserted for arterial pressure monitoring and blood gas analysis. One-lung ventilation will be achieved using a left-sided double-lumen tube.

Study Interventions

Combined ESPB and INB Group

A single-injection ultrasound-guided erector spinae plane block will be performed at the T7 vertebral level before surgery. After confirmation of needle position with a saline test injection, 30 mL of 0.375% ropivacaine will be injected into the fascial plane beneath the erector spinae muscle.

At the end of surgery, intercostal nerve blocks will be performed under direct thoracoscopic visualization. Ropivacaine 0.375% will be administered using 4 mL at the fifth intercostal space and 3 mL at the sixth, seventh, or eighth intercostal spaces according to port location.

INB Group

Patients assigned to the INB group will receive surgical intercostal nerve blocks identical to those administered in the EI group but without ESPB.

Surgical Procedure

Thoracoscopic surgery will be performed using a three-port technique. A chest tube will be inserted through the largest incision at the completion of surgery.

Postoperative Analgesia

Intravenous patient-controlled analgesia will be initiated immediately after surgery. The PCA solution will contain fentanyl, morphine, ketorolac, and ramosetron. The pump will be programmed with a basal infusion rate of 1 mL/h, a bolus dose of 1 mL, and a lockout interval of 15 minutes.

Ketorolac suppositories (15 mg) will be administered as rescue analgesia when pain is inadequately controlled.

Outcome Assessment

Pain intensity will be assessed using an 11-point numerical rating scale (0–10) at 1, 2, 3, 4, 5, 6, 24, and 48 hours after surgery by nurses blinded to treatment allocation.

Patients will be instructed to perform deep-breathing exercises using a three-ball incentive spirometer, and inspiratory volumes will be recorded.

Arterial blood gas analyses will be obtained on postoperative days 1 and 2.

Outcome Measures

Primary Outcome

- Numerical Rating Scale (NRS) pain score at 1 hour postoperatively.

Secondary Outcomes

- Numerical rating scale pain scores at individual postoperative time points.
- Total opioid consumption during the first 48 postoperative hours.
- Requirement for rescue analgesia.
- Incentive spirometry volumes at 24 and 48 hours.
- Arterial oxygen tension (PaO_2) and carbon dioxide tension ($PaCO_2$) on postoperative days 1 and 2.
- Complications related to ESPB or intercostal nerve block, including pneumothorax, local anesthetic systemic toxicity, vasoplegia, low cardiac output syndrome, respiratory depression, reintubation, and cardiac arrest.

Sample Size Calculation

Based on a previous study reporting a mean maximum postoperative pain score of 6.16 ± 1.95 following VATS with intercostal nerve block, a sample size of 27 patients per group will provide 80% power to detect a 25% reduction in pain scores at a two-sided significance level of 0.05. To allow for potential dropouts, 30 patients will be enrolled in each group.

Statistical Analysis

Continuous variables will be analyzed using Student’s t-test or the Mann–Whitney U test, as appropriate. Categorical variables will be compared using the chi-square test or Fisher’s exact test.

Repeated postoperative pain scores will be analyzed using a mixed-effects model. Opioid consumption, arterial blood gas values, rescue analgesic requirements, and block-related complications will also be compared between groups.

Data will be presented as mean (standard deviation), median (interquartile range), or frequency (%). A two-sided p value < 0.05 will be considered statistically significant.

Statistical analyses will be performed using R version 3.5.1 (R Foundation for Statistical Computing, Vienna, Austria) and Python version 3.7 (Python Software Foundation, Wilmington, DE, USA).
