# Supplementary material for: Erector spinae plane block added to intercostal nerve block versus intercostal nerve block alone for postoperative analgesia in video-assisted thoracoscopic surgery: A randomized controlled trial
Source: PLoS One. 2026 Oct 27;21(10):e0361408. doi: 10.1371/journal.pone.0361408 (PMC13712513; doi:10.1371/journal.pone.0361408)
Supplement: Supplementary file 3 [file pone.0361408.s003.docx]

**S3 Table.** Time-specific between-group contrasts in postoperative NRS pain scores from the linear mixed-effects model

| **Postoperative time** | **Estimated between-group difference (EI - INB)** | **95% CI** | **Holm-adjusted  p-value** |
| --- | --- | --- | --- |
| 1 h | 0.31 | −0.98 to 1.60 | 1.000 |
| 2 h | −0.52 | −1.80 to 0.77 | 1.000 |
| 3 h | −1.10 | −2.38 to 0.18 | 0.738 |
| 4 h | −0.87 | −2.15 to 0.42 | 1.000 |
| 5 h | −0.73 | −2.02 to 0.55 | 1.000 |
| 6 h | −0.25 | −1.53 to 1.03 | 1.000 |
| 24 h | −0.38 | −1.67 to 0.90 | 1.000 |
| 48 h | −0.65 | −1.94 to 0.65 | 1.000 |

The prespecified primary 1-hour comparison was analyzed separately using Student's t-test without multiplicity adjustment; the 1-hour value in this table represents the post hoc LMM-derived contrast and does not replace the primary analysis. EI, combined erector spinae plane block and intercostal nerve block; INB, intercostal nerve block; NRS, Numerical Rating Scale; CI, confidence interval; LMM, linear mixed-effects model.
